# Protein regulation strategies of the mouse spleen in response to *Babesia microti* infection

**DOI:** 10.1186/s13071-020-04574-5

**Published:** 2021-01-19

**Authors:** Xiaomin Xue, Shuguang Ren, Xiaohong Yang, Abolfazl Masoudi, Yuhong Hu, Xiaoshuang Wang, Hongxia Li, Xiaojing Zhang, Minjing Wang, Hui Wang, Jingze Liu

**Affiliations:** 1 Hebei Key Laboratory of Animal Physiology, Biochemistry and Molecular Biology, College of Life Sciences, Hebei Normal University, Shijiazhuang, 050024 Hebei People’s Republic of China; 2 The Fourth Hospital of Hebei Medical University, Shijiazhuang, 050011 Hebei People’s Republic of China; 3 Department of Pathogenic Biology, College of Basic Medicine, Hebei Medical University, Shijiazhuang, 050017 Hebei People’s Republic of China; 4 Instrumental Analysis Center, Hebei Normal University, Shijiazhuang, 050024 Hebei People’s Republic of China

**Keywords:** *Babesia microti*, Spleen, Quantitative proteomics, Phosphorylation, Immune

## Abstract

**Background:**

*Babesia* is a protozoan parasite that infects red blood cells in some vertebrates. Some species of *Babesia* can induce zoonoses and cause considerable harm. As the largest immune organ in mammals, the spleen plays an important role in defending against *Babesia* infection. When infected with *Babesia*, the spleen is seriously injured but still actively initiates immunomodulatory responses.

**Methods:**

To explore the molecular mechanisms underlying the immune regulation and self-repair of the spleen in response to infection, this study used data-independent acquisition (DIA) quantitative proteomics to analyse changes in expression levels of global proteins and in phosphorylation modification in spleen tissue after *Babesia microti* infection in mice.

**Results:**

After mice were infected with *B. microti*, their spleens were seriously damaged. Using bioinformatics methods to analyse dynamic changes in a large number of proteins, we found that the spleen still initiated immune responses to combat the infection, with immune-related proteins playing an important role, including cathepsin D (CTSD), interferon-induced protein 44 (IFI44), interleukin-2 enhancer-binding factor 2 (ILF2), interleukin enhancer-binding factor 3 (ILF3) and signal transducer and activator of transcription 5A (STAT5A). In addition, some proteins related to iron metabolism were also involved in the repair of the spleen after *B. microti* infection, including serotransferrin, lactoferrin, transferrin receptor protein 1 (TfR1) and glutamate-cysteine ligase (GCL). At the same time, the expression and phosphorylation of proteins related to the growth and development of the spleen also changed, including protein kinase C-δ (PKC-δ), mitogen-activated protein kinase (MAPK) 3/1, growth factor receptor-bound protein 2 (Grb2) and P21-activated kinase 2 (PAK2).

**Conclusions:**

Immune-related proteins, iron metabolism-related proteins and growth and development-related proteins play an important role in the regulation of spleen injury and maintenance of homeostasis. This study provides an important basis for the diagnosis and treatment of babesiosis.
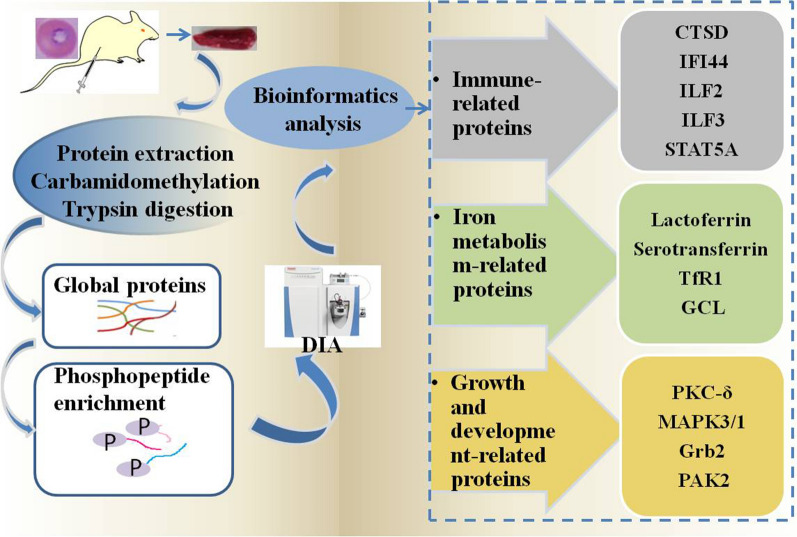

## Background

*Babesia* is a blood parasitic protozoan [1]. Hosts infected with *Babesia* develop babesiosis. *Babesia* is mainly transmitted by ticks and sometimes through blood transfusion or pregnancy [2, 3]. People with *Babesia* infection exhibit symptoms such as fever, anaemia and haemoglobinuria, while people with severe infections may suffer multiple organ damage [4]. The life cycle of *Babesia microti* involves two hosts, including some species of *Ixodes* and the white-footed mouse (*Peromyscus leucopus*), and *B. microti* can also sometimes parasitize humans [5, 6].

The spleen is the largest immune system organ in the body. It is very important for the clearance of parasites. People with splenic insufficiency or who have undergone splenectomy will experience more severe diseases after suffering from babesiosis [7]. *Babesia* infection results in severe spleen injury. However, the injured spleen will still actively initiate immunomodulatory responses during self-repair. The spleen contains a large number of B lymphocytes and macrophages and can also produce immunoglobulins and complement factors that exert immune functions [8]. Initiation and operation of these immunomodulatory functions and repair mechanisms of the spleen are achieved through coordination among many proteins. These proteins exert their functions through changes in their expression levels and post-translational modifications. To elucidate the molecular regulatory mechanisms of immune responses and self-repair exhibited by the spleen during different stages of *Babesia* infection, this study used data-independent acquisition (DIA) [9] quantitative proteomics to comprehensively analyse changes in expression levels and phosphorylation modifications of proteins in mouse spleen tissues during different stages of *B. microti* infection. To our knowledge, this is the first proteomics study on mammalian spleen after *Babesia* infection. This study showed that expression levels of immune-, iron metabolism- and growth and development-related proteins in mouse spleen tissues changed after *Babesia* infection. In addition, phosphorylation modifications of these proteins also changed. These study results may provide a theoretical basis for analyses of how the spleen perceives *Babesia* infection stress and resists *Babesia* infection and a theoretical basis for effective detection, diagnosis and treatment of babesiosis.

## Methods

### Dissection of mouse spleen and sample preparation

*Babesia microti* (ATCC PRA-99™) was obtained from the Institute of Laboratory Animal Sciences, Chinese Academy of Medical Sciences. Female BALB/c mice were raised to 6 weeks of age, and then the mice received intraperitoneal injection of 150 μl of *B. microti*-infected erythrocytes (1.8 × 10^7^) [10]. After 5 days, 8 days, 11 days and 19 days, mice infected with *B. microti* were anaesthetized and killed. Blood was collected from the tail tip every day to create blood smears. After Giemsa staining, *B. microti* infection status was observed under a microscope, and the parasitaemia level was calculated. The spleen of each mouse was dissected for total protein and quantitative phosphorylation proteomics experiments. The spleens of four mice were used for the uninfected group and each infection period. Four biological repeats were carried out, and a total of 20 mice were used. Spleen tissue samples (2 mm^3^) were immediately placed in 4% glutaraldehyde fixative for storage and used for subsequent transmission electron microscope (TEM) analysis. The remaining tissues were rapidly frozen at – 80 °C for subsequent proteomics studies. All experimental procedures were approved by the Animal Ethics Committee of Hebei Normal University (no. 165031). The involved animal feeding and material collection procedures were all performed in the level 2 biosafety laboratory in The Fourth Hospital of Hebei Medical University.

### Preparation of TEM samples and observation

Fresh spleen tissues were fixed in 4% glutaraldehyde (Alfa Aesar, Germany) phosphate buffer (pH 7.4) at 4 °C for more than 4 h. After flushing with 0.1 M of phosphate buffer (pH 7.4), the tissues were fixed again in 1% buffered osmium tetroxide solution (SPI-CHEM, USA) for 2 h. In the next stage, the samples were washed in phosphate buffer and dehydrated in an acetone series. Dehydrated tissues were embedded in epoxy resin (SPI-VHEM, USA). Resin polymerization was conducted at 60 °C for 36 h. The ultrathin sections (50–60 nm) were stained with uranyl acetate (Polyscience, USA) and citrate lead (Sigma Aldrich, USA). The ultrastructure was observed by TEM (Hitachi H7650, Japan).

### Protein extraction and digestion

Spleen tissues from different periods were ground (1 M pH 6.8) in a mortar containing a protease inhibitor cocktail (Roche, Mannheim, Germany) and centrifuged (4 °C, 12,000×*g*, 15 min). The supernatant was collected, and Tris-saturated phenol (pH 7.8) was added, followed by centrifugation (4 °C, 12,000×*g*, 15 min). After removing the supernatant, an equal volume of 50 mM Tris-HCl (pH 8.0) was added, followed by centrifugation (4 °C, 12,000×*g*, 20 min). After removing the supernatant, 0.1 M ammonium acetate was added to precipitate the protein at – 20 °C overnight. The mixture was centrifuged (4 °C, 12,000×*g*, 20 min), the protein pellet was washed with methanol twice, and the extracted proteins were lyophilized and stored at – 80 °C. The protein samples were subjected to alkylation for cysteine carbamidomethylation. After the protein was digested with trypsin (1:20 w/w, Promega, USA), the peptides were desalted with C18 SPE (CNW^®^, China) according to the manufacturer’s instructions. The concentrations of the peptides obtained after trypsin digestion were determined using a BCA Protein Assay kit (Pierce Biotechnology). After normalizing the concentrations of the samples, the enzyme efficiency was monitored by LC–MS [consists of a UPLC M-Class system (Waters, USA) and a Q Exactive HF (Thermo Fisher, USA) mass spectrometer].

### Phosphopeptide enrichment

**Fig. 1:**
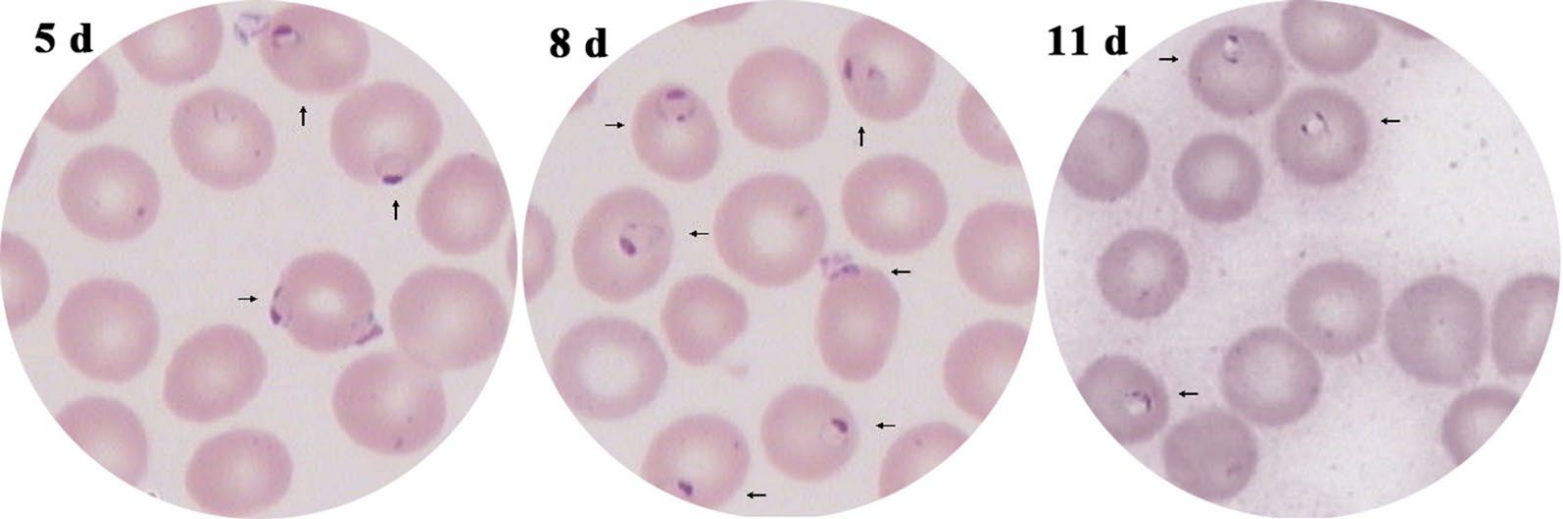
Observation of red blood cells from mice infected with *B. microti*. Giemsa blood smear staining of *B. microti* in red blood cells. *B. microti* is indicated by arrows

The workflow for phosphopeptide enrichment and quantitative analysis is shown in Fig. 1. Aliquots of TiO_2_ (GL Sciences, Japan) beads were washed three times using buffer with 50% acetonitrile (ACN) containing 2% trifluoroacetic acid (TFA), saturated with glutamic acid. The TiO_2_ beads and peptides were dissolved in 800 µl of the same buffer and gently shaken at room temperature for 1 h. The TiO_2_ beads were then washed with 50% ACN to remove the non-phosphorylated peptides. The TiO_2_ beads were washed twice with 50% ACN containing 20 mM ammonium acetate. Phosphopeptides were then eluted from the TiO_2_ beads with 200 µl of 0.3 M NH_4_OH one time and with 200 µl of 0.5 M NH_4_OH two times. The enriched phosphopeptides were then lyophilized and frozen at – 20 °C for subsequent use.

### High-pH RP-HPLC for peptide separation

Each sample comprised an equal mixture of digested peptides. The mixture was then separated into ten eluted components by high-pH reversed phase high-performance liquid chromatography (RP-HPLC) (Waters e2695, USA) through a Durashell C18 column (5-μm particle size, 100-Å pore size, 4.6 mm × 250 mm, Agela, China). The liquid phase separation gradient was as follows: 2% solvent B (100% ACN containing 5 mM ammonium formate, pH 10.0) and 98% solvent A (100% water containing 5 mM ammonium formate, pH 10.0) for 10 min, followed by 2–50% solvent B in 70 min, at a flow rate of 1 ml/min using a linear gradient. Each elution component was collected every 1 min, and a total of 60 elutions were collected. Every six elutions were mixed together (i.e., 1, 11, 21, 31, 41, 51). Then, the samples were dried and stored at – 80 °C.

### Data-dependent acquisition (DDA) spectral library construction

DDA spectral library construction was performed as previously described [11]. Briefly, groups of ten samples were separated by liquid chromatography, then resuspended in a 0.1% formic acid water solution containing iRT reagent (Spectronaut, Switzerland) and further analysed by LC–MS [consists of a UPLC M-Class system (Waters Corp., Milford, MA, USA) and Q Exactive HF (Thermo Fisher, USA) mass spectrometer]. Each sample was first loaded onto a C18 RP trap column (5-μm particle size, 100-Å pore size, 180-μm ID × 20-mm length; Waters Corp.) and then separated on a C18 RP analytical column (1.8-μm particle size, 100-μm ID × 150-mm length; Waters Corp.] at a flow rate of 300 nl/min using a linear ACN gradient of 2–8% solvent B in 6 min and then 8–35% solvent B in next 114 min (solvent A: 99.9% H_2_O, 0.1% formic acid; solvent B: 99.9% ACN, 0.1% formic acid). The sample was electrosprayed into the Q Exactive HF (2.0 kV and 290 °C). The Q Exactive HF parameters were set as previously described [11]. The process of DDA for enrichment of phosphopeptides was the same as described above. Proteome Discoverer (version 2.2, Thermo Fisher Scientific) was used to search the DDA mass spectrometry results for the above groups of ten samples to construct DDA spectral libraries. The database was derived from the protein sequences for the Mus musculus downloaded from UniProt (2017/12/07, 16944 sequences), and trypsin, human keratins and *Babesia* sequences were used to establish a contaminated database. The data search parameters were set as previously described [11].

### Data-independent acquisition (DIA) spectral acquisition and data analysis

DIA analysis was carried out for each sample. The chromatographic conditions for DIA were the same as those for DDA spectral library construction. The DIA mass spectrometry parameters were as follows: (i) DIA mode; scanning range for a full scan, 350–1200 *m*/*z*; resolution of the precursor ion, 60,000; automatic gain control (AGC) target, 3 × 10^6^; maximum ion injection time (maximum IT), 50 ms; (ii) HCD normalized collision energy, 27%; (iii) DIA MS2 scanning, 34 consecutive windows, each of which was set to 26 *m*/*z* and a 1 *m*/*z* overlap between two adjacent windows; (iv) MS2 scan resolution, 30,000; AGC target, 1 × 10^6^; maximum IT, set to auto.

DIA findings were analysed using Spectronaut software (version 11.0, Switzerland). The default parameters for DIA data analysis were used, where the false discovery rate (FDR) for proteins and peptides was set to < 1%. The protein expression levels in all treatment groups were compared with the control group, and the ratio was regarded as the change in protein or peptide expression, which was also the basis for further data analysis and discussion. Proteins quantified with at least two unique peptides and whose *Q* value was < 0.05 and expression change times > 1.5 were considered to have significant changes in expression. For phosphopeptides, we only focused on the quantitative phosphopeptide results. If the change in phosphopeptides was > 1.5 times, the degree of phosphorylation modifications was considered changed.

### Bioinformatics analysis

Bioinformatics analysis was performed for all differentially expressed proteins or phosphopeptides. Proteins with similar expression characteristics were clustered with GProX [12]. The number of clusters was set to 4, and a fixed regulation threshold (an upper limit of 0.58 for protein upregulation and a lower limit of − 0.58 for protein downregulation, corresponding to the original ratios 1.5 and 0.67, respectively) was used. Principal component analysis (PCA) was performed with online analysis software (http://www.omicsolution.org/wu-kong-beta-linux/main/). PANTHER software (http://pantherdb.org/) was used for Gene Ontology (GO) functional categories. Pathways associated with the differentially expressed proteins were identified using the Kyoto Encyclopedia of Genes and Genomes (KEGG) database (http://www.kegg.jp/kegg/). The tool used for KEGG analysis was KEGG Mapper (https://www.kegg.jp/kegg/tool/map_pathway2.html).

### Parallel reaction monitoring (PRM) mass spectrometry and data analysis

To verify the protein expression levels obtained by DIA, ten selected proteins were further quantified by PRM. One signature peptide without missing sites was selected based on DIA data, and each peptide that we selected belonged only to one specific target protein. PRM analysis was performed in the Q-Exactive HF mass spectrometer (Thermo Fisher, USA) with the same LC gradient settings as above. The parameters were set as follows: (i) resolution of the full scan: 60,000; (ii) AGC target: 3e6; (iii) full scan range: 350–1200 *m*/*z*; (iv) maximum IT: 50 ms; (v) MS2 scan resolution: 30,000; (vi) AGC target: 1e5; (vii) maximum IT: 100 ms. The mass spectrometry proteomics data were analysed using Skyline (MacCoss Laboratory, University of Washington) [13]. We assessed and corrected the peak according to the transitions, retention time, mass accuracy and MS/MS spectra. The area under the curve (AUC) of each transition was extracted and exported to Excel for further data analysis.

## Results

### Observation of red blood cells from mice infected with *B. microti* under a microscope

The results showed that parasitaemia levels in red blood cells after infection reached the highest proportion (nearly 50%) at 8 days, and the parasitaemia levels substantially decreased at 11 days after infection (nearly 10%) (Fig. 1). At 19 days after infection, *B. microti* was not observed in red blood cells.

### Effects of *B. microti* infection on the spleen

**Fig. 2:**
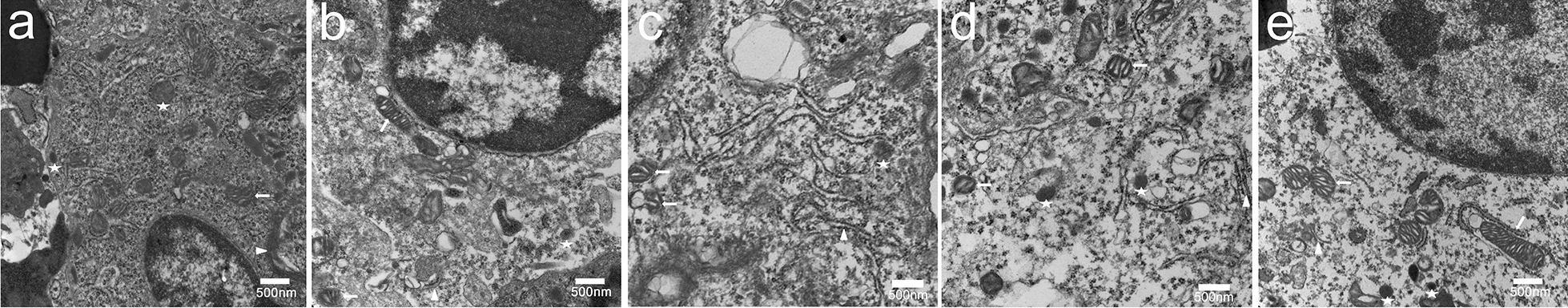
TEM observation of splenocytes in mice infected with *B. microti* at different stages. **a** 0 day (control); **b** 5 days; **c** 8 days; **d** 11 days; **e** 19 days. The number of mitochondria in mouse splenocytes infected by *B. microti* was significantly decreased. On the 8th day, the endoplasmic reticulum was obviously dilated, which was accompanied by degranulation. On the 11th day, the numbers of lysosomes increased, and the endoplasmic reticulum was still dilated, which was accompanied by degranulation. By the 19th day after infection, the expansion of the endoplasmic reticulum was significantly reduced, the degranulation was also weakened, and the number of lysosomes returned to the normal level. The short arrow points to mitochondria, the pentagram to the lysosome, the triangle to the endoplasmic reticulum and long scissors to autophagy

The spleens of the mice infected by *B. microti* became enlarged, and some organelles were abnormal. Gross morphological changes in the spleen at different time points after *B. microti* infection are shown in Additional file 1: Figure S1. Changes in the subcellular structure in mice are shown in Fig. 2. As shown in the figure, the endoplasmic reticulum of splenocytes from uninfected mice showed normal morphological characteristics, with the largest number of mitochondria per unit area (14 mitochondria can be observed in the visual field of the same area) and a small number of lysosomes per unit area (4 lysosomes can be observed in the visual field of the same area). At 5 days after infection, the endoplasmic reticulum of spleen cells showed slight dilation and slight degranulation. At this time, the number of mitochondria per unit area decreased significantly (only 4 mitochondria could be observed in the visual field), and the number of lysosomes per unit area was still small (4 lysosomes could be observed in the visual field). Furthermore, the endoplasmic reticulum of spleen cells was obviously dilated and severely degranulated at 8 days after infection; at this time, the number of mitochondria per unit area was reduced (2 mitochondria could be observed in the visual field), and the number of lysosomes per unit area was still small (2 lysosomes could be observed in the visual field). At this time, autophagosomes appeared. Splenomegaly was most obvious at 11 days after infection (Additional file 1: Figure S1), and the endoplasmic reticulum was still dilated and degranulated. In addition, the number of mitochondria per unit area was still small (3 mitochondria could be observed in the visual field), the number of lysosomes was the greatest (9 lysosomes could be observed in the visual field), and the presence of autophagosomes remained evident during this period. At 19 days after infection, the size of the spleen returned to almost the same as that at 5 days after infection, and the endoplasmic reticulum in spleen cells basically returned to a normal morphology, while the number of mitochondria per unit area was still small (5 mitochondria could be observed in the visual field). At this time, the number of lysosomes decreased (3 lysosomes could be observed in the visual field), and autophagosomes were no longer observed.

### Identification and quantification of global proteins and phosphopeptides

**Fig. 3:**
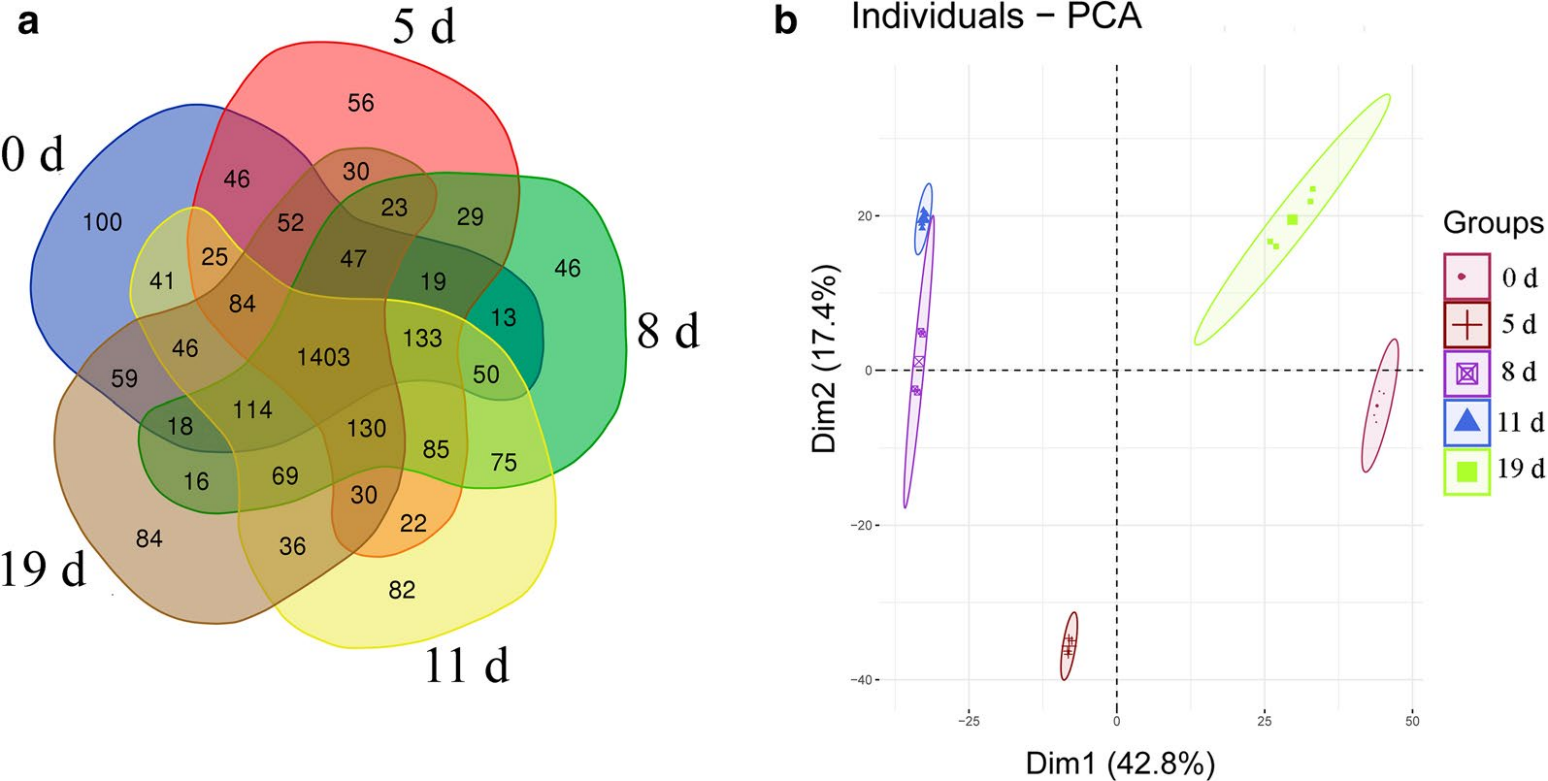
Statistics for the identified global proteins. **a** PCA of global protein mass spectrometry data at the five sampling times. **b** Venn diagram of proteins identified in various stages (global proteins)

Changes in global protein expression levels and phosphorylation modification in the spleens of mice infected with *B. microti* were analysed by DIA quantitative proteomics. The whole experimental design is shown in Additional file 1: Figure S1. The numbers of identified proteins in mice in the uninfected group (0 day) and after 5 days, 8 days, 11 days and 19 days of *B. microti* infection were 2804, 2890, 2888, 2936 and 2918, respectively; the numbers of proteins that had a coefficient of variation (CV) value < 20% among four biological repeats in all groups were 2250, 2214, 2270, 2425 and 2241, respectively. PCA was performed on data in four repeats for these five periods (Fig. 3a). The figure shows that the similarity among repeated data in all groups was high, indicating that the reproducibility of the data in four repeats was high, while the five groups of data for the different infection periods were significantly different. The identified results were subject to Venn diagram analysis (Fig. 3b). The number of proteins that were identified in all five periods was 1403, of which 966 proteins were differentially expresses. The information for intersecting proteins is provided in Additional file 2: Dataset S1. The mass spectrometry proteomics data have been deposited in the ProteomeXchange Consortium via the iProX partner repository (accession no. IPX0002204000/PXD019236).

**Fig. 4:**
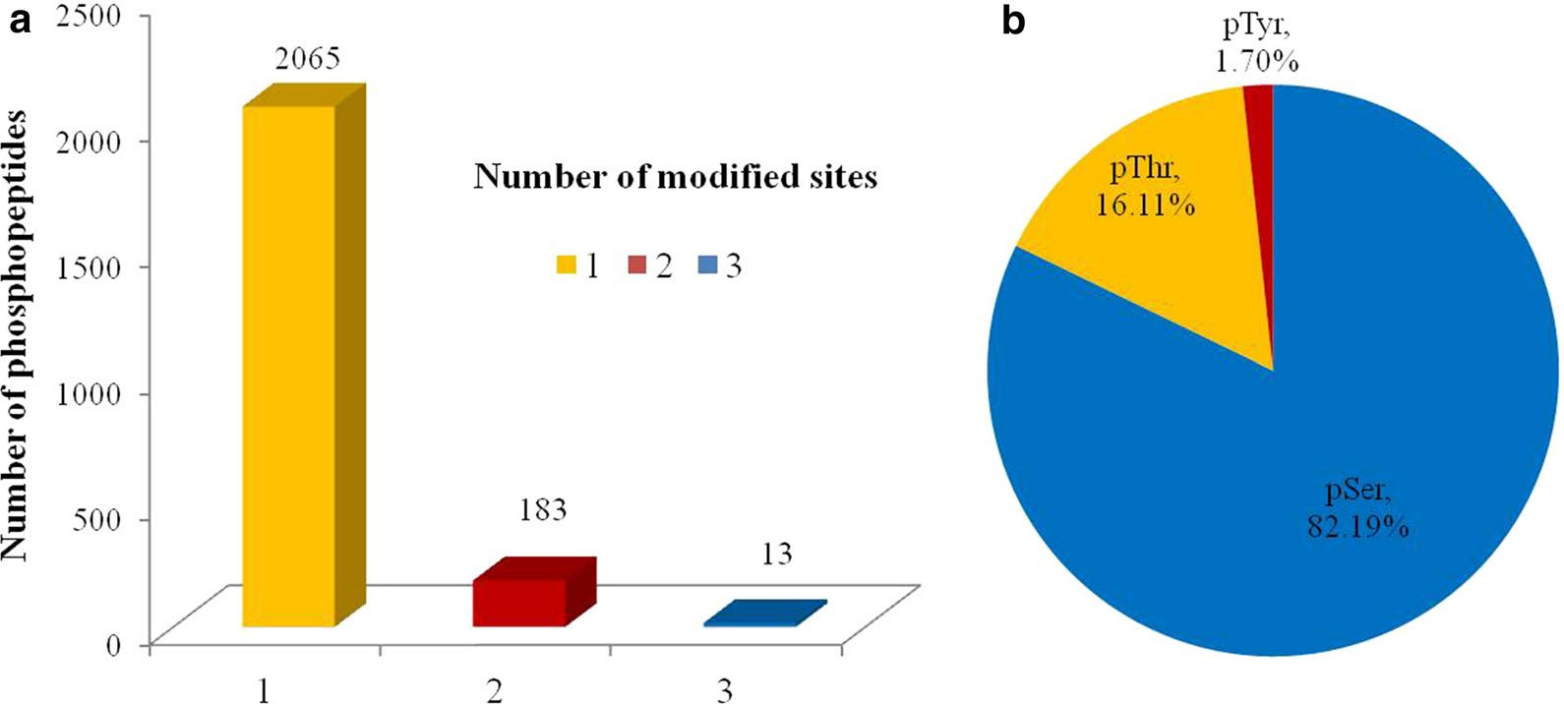
Statistics for the identified phosphorylated peptides. **a** Statistical analysis of the number of modification sites on phosphorylated peptide fragments. **b** Statistical analysis of percentages of the three types of amino acid phosphorylation

The quantitative results for phosphopeptides in all groups identified by mass spectrometry were analysed using Spectronaut 11 software. The numbers of identified phosphopeptides in mice in the uninfected group (0 day) and after 5 days, 8 days, 11 days and 19 days of *B. microti* infection were 12,373, 12,689, 12,792, 12,670 and 12,501, respectively; the numbers of phosphopeptides with a CV value < 20% among four biological repeats in all groups were 5363, 8638, 9746, 6725 and 8042, respectively. PCA was performed on data from four repeats for these five periods, and the Venn diagram shows 2261 intersecting phosphopeptides in the five periods (Additional file 1: Figure S2). These 2261 phosphopeptides included 2470 phosphorylation modification sites, of which 2065 peptides contained 1 phosphorylation site, 183 peptides contained 2 phosphorylation sites, and 13 peptides contained 3 phosphorylation sites (Fig. 4a). Among 2470 phosphorylation sites, 82.19% occurred at serine residues, 16.11% occurred at threonine residues, and 1.70% occurred at tyrosine residues (Fig. 4b). When the mass spectrometry identification results of the same polypeptide in different periods had a fold change > 1.5, the peptide was considered to have differentially changed; in other words, the level of phosphorylation modification at this site changed. A total of 2169 phosphopeptides, corresponding to 1011 proteins, showed differences in mass spectrometry identification results. The information for intersecting phosphopeptides is provided in Additional file 2: Dataset S2. The mass spectrometry proteomics data have been deposited in the ProteomeXchange Consortium via the iProX partner repository (accession no. IPX0002209000/PXD019319).

### Cluster analysis and GO annotation analysis of differentially expressed proteins

**Fig. 5:**
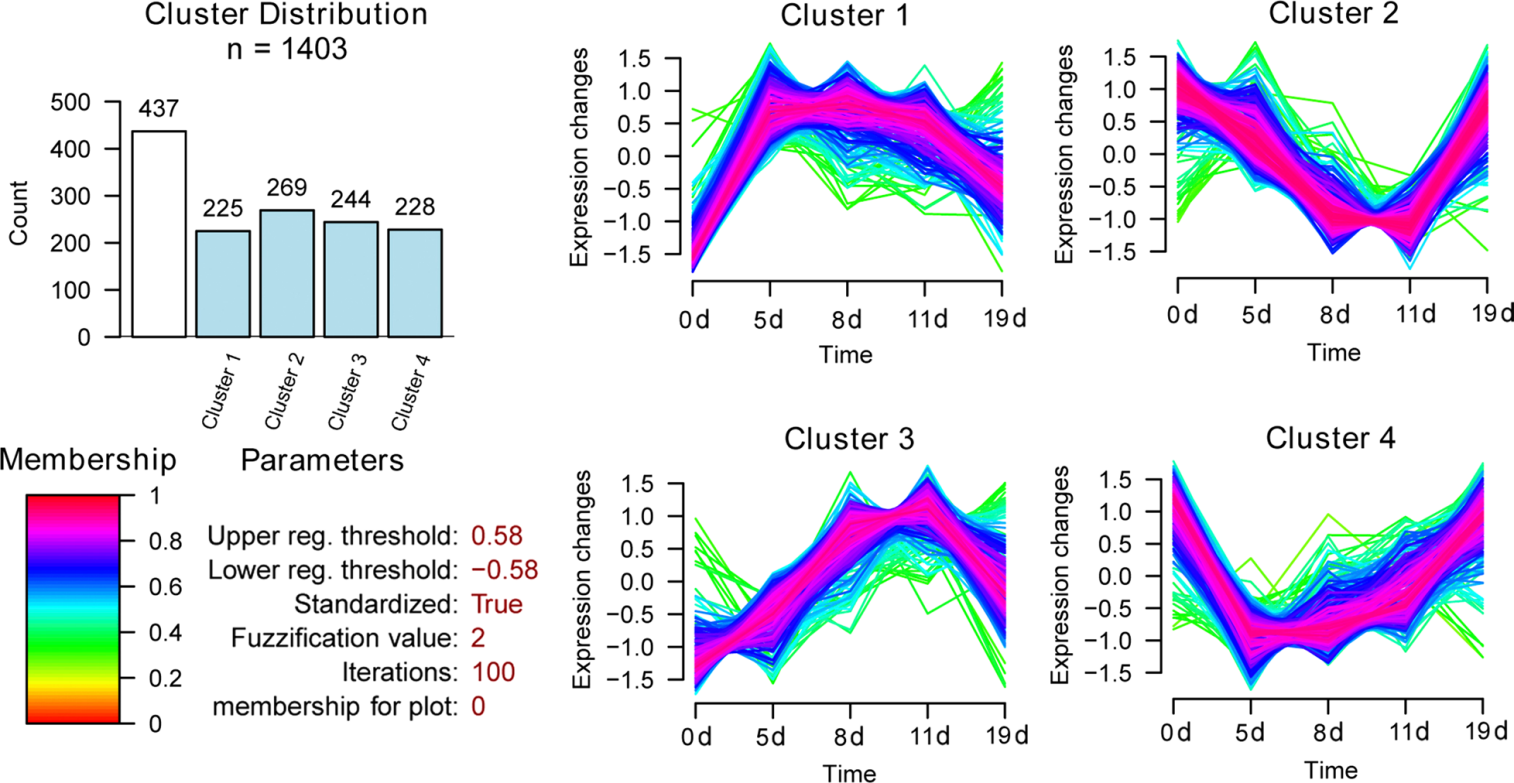
Cluster analysis of differentially expressed proteins in the spleen tissues of mice infected with *B. microti*. A log2 value > 0.58 indicated upregulation, showing that this protein was upregulated, while a log2 value < − 0.58 indicated downregulation, showing that this protein was downregulated

All differentially expressed proteins were classified by cluster analysis and GO annotation analysis, which provided information for further analysis of signalling pathways. For the periods of 5 days/0 day, 8 days/0 day, 11 days/0 day and 19 days/0 day of *B. microti* infection, cluster analysis was performed on 1403 intersecting proteins that had annotation information (Fig. 5). The results showed that Cluster 0 had 437 proteins with differential expression level changes that were not significant. Cluster 1 had 225 proteins, and the expression levels of these proteins were upregulated with increasing parasitaemia levels; however, the expression levels of these proteins slightly decreased with recovery at 19 days. Functions of these proteins were associated with defending against *B. microti* infection. Cluster 2 had 269 proteins. The expression levels of these proteins were downregulated at 5 days, 8 days and 11 days after infection and upregulated at 19 days. Cluster 3 had 244 proteins. The expression levels of these proteins were upregulated at 5 days, 8 days and 11 days after infection and downregulated to normal levels at 19 days. Cluster 4 had 228 proteins. Their expression levels were downregulated at 5 days after infection and gradually upregulated at 8 days, 11 days and 19 days.

**Fig. 6:**
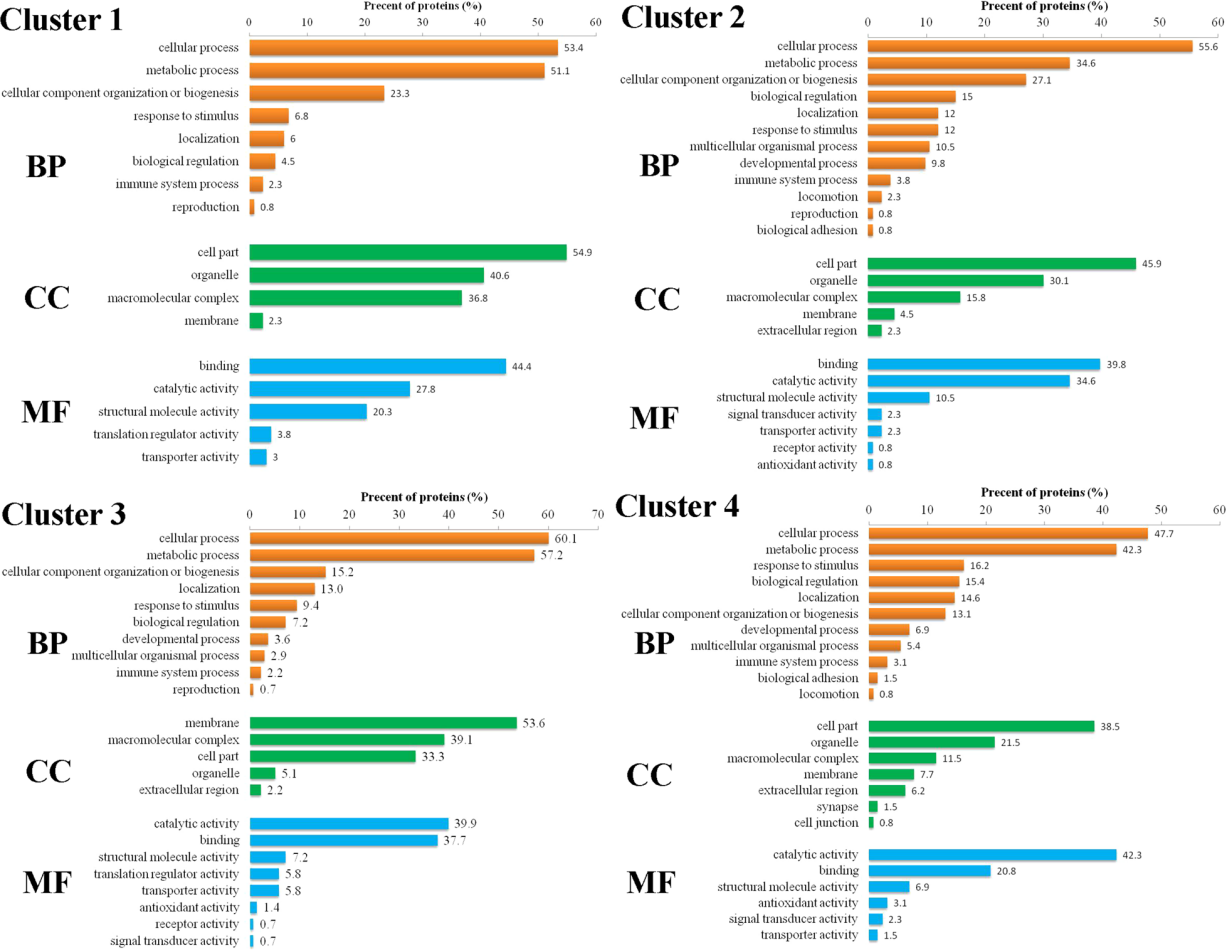
GO functional annotations of differentially expressed proteins in four clusters. Biological Process, Cellular Component and Molecular Function (orange, green and blue bars, respectively)

Proteins in Cluster 1–Cluster 4 that exhibited expression changes were subject to GO annotation analysis (Fig. 6). These proteins were classified into the following categories: Biological Process, Cellular Component and Molecular Function (orange, green and blue bars, respectively). Among the four clusters, cellular process and metadata process were the most abundant nodes under the Biological Process node, and binding and catalytic activity were the most abundant nodes under the Molecular Function node. However, cell part and organelle process were the most abundant nodes under the Cellular Component node in Cluster 1, Cluster 2 and Cluster 4. By comparison, membrane and macromolecular complex processes were the most abundant nodes under the Cellular Component node in Cluster 3.

### Cluster analysis and GO enrichment analysis of differentially expressed phosphopeptides

Phosphorylation is one of the most important post-translational modifications that regulate biological functions. All the phosphorylated peptides with different changes were classified to create conditions for further analysis of signalling pathways by cluster analysis and GO annotation analysis.

For the periods of 5 days/0 day, 8 days/0 day, 11 days/0 day and 19 days/0 day of *B. microti* infection, cluster analysis was performed on differentially modified phosphopeptides (Additional file 1: Figure S3). These proteins were grouped into five clusters. The results showed that the phosphorylation modification changes in 92 peptides in Cluster 0 were not significant. The phosphorylation modification levels of 560 phosphopeptides in Cluster 1 were upregulated with increasing parasitaemia levels. However, the modification levels were downregulated at 11 days when the parasitaemia levels decreased and then gradually returned to normal levels in the recovery period at 19 days. The modification levels of 547 phosphopeptides in Cluster 2 were downregulated at 5 days, 8 days and 11 days after *B. microti* infection and upregulated at 19 days. The phosphorylation modification levels of 408 phosphopeptides in Cluster 3 were upregulated at 5 days and 8 days and returned to normal levels at 11 days and 19 days. The phosphorylation modification levels of 554 phosphopeptides in Cluster 4 were downregulated at 5 days, 8 days and 11 days and upregulated again at 19 days.

GO annotation was performed on proteins corresponding to differentially expressed phosphopeptides in Cluster 1–Cluster 4 (Additional file 1: Figure S4). These proteins were classified into the following categories: Biological Process, Cellular Component and Molecular Function (orange, green and blue bars, respectively). Among the four clusters, cellular process and metadata process were the most abundant nodes under the Biological Process node, and binding and catalytic activity were the most abundant nodes under the Molecular Function node. In addition, cell part and organelle were the most abundant nodes under the Cellular Component node.

### KEGG pathway analysis of differentially expressed proteins and phosphorylated proteins

**Fig. 7:**
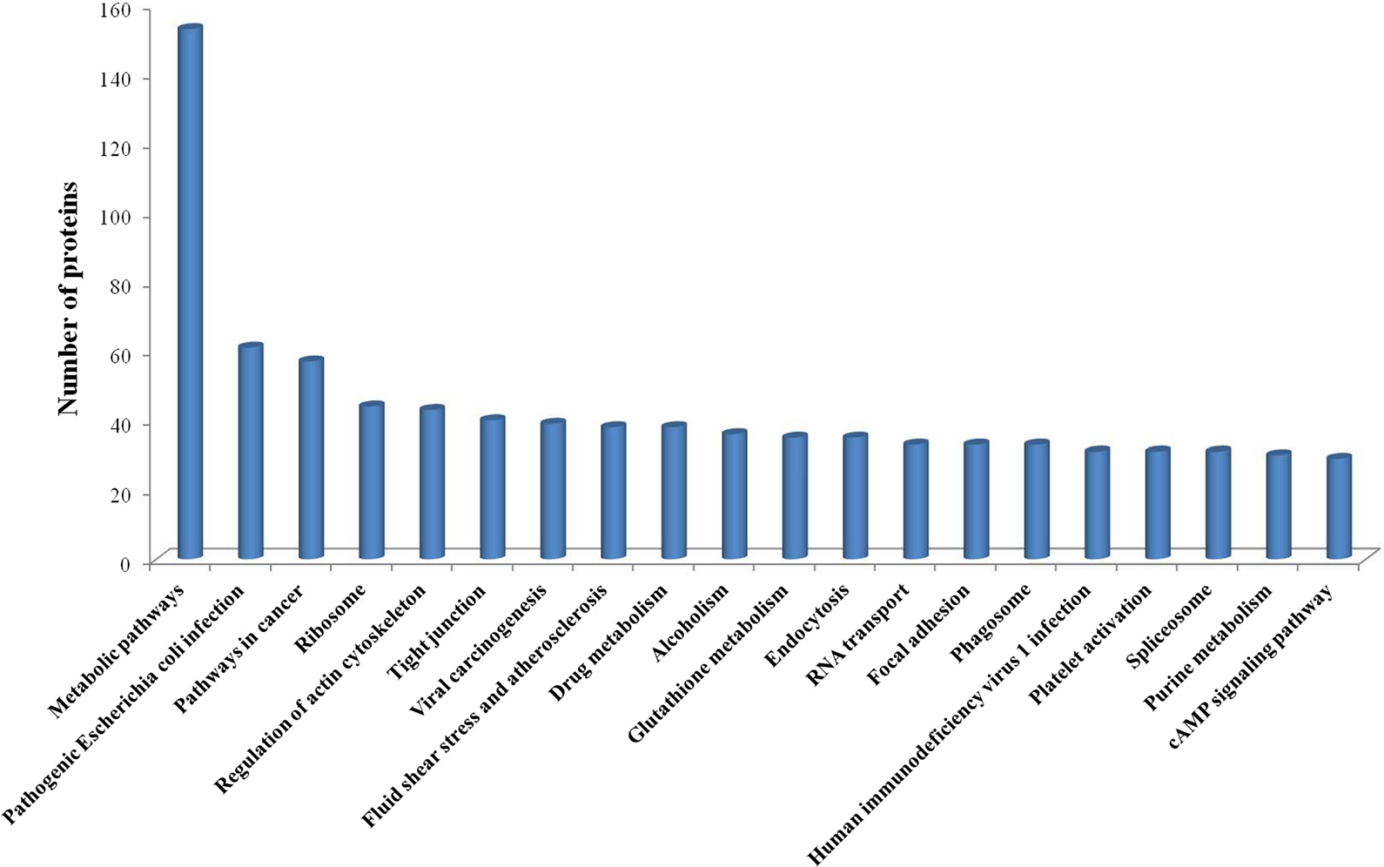
KEGG pathway analysis of differentially expressed proteins

Regulatory pathways associated with differentially expressed proteins were identified, and their roles in host defence against *Babesia* infection were determined by analysing KEGG signalling pathways. A total of 966 differentially expressed proteins from Cluster 1–Cluster 4 were analysed for signalling pathway analysis (Fig. 5). A total of 288 signalling pathways were involved (Fig. 7); 153 proteins were involved in metabolic pathways, and 44 proteins were involved in ribosome biogenesis. The following pathways were also involved: the mitogen-activated protein kinase (MAPK) signalling pathway (ko04010), T cell receptor signalling pathway (ko04660) and Amoebiasis pathway (ko05146).

**Fig. 8:**
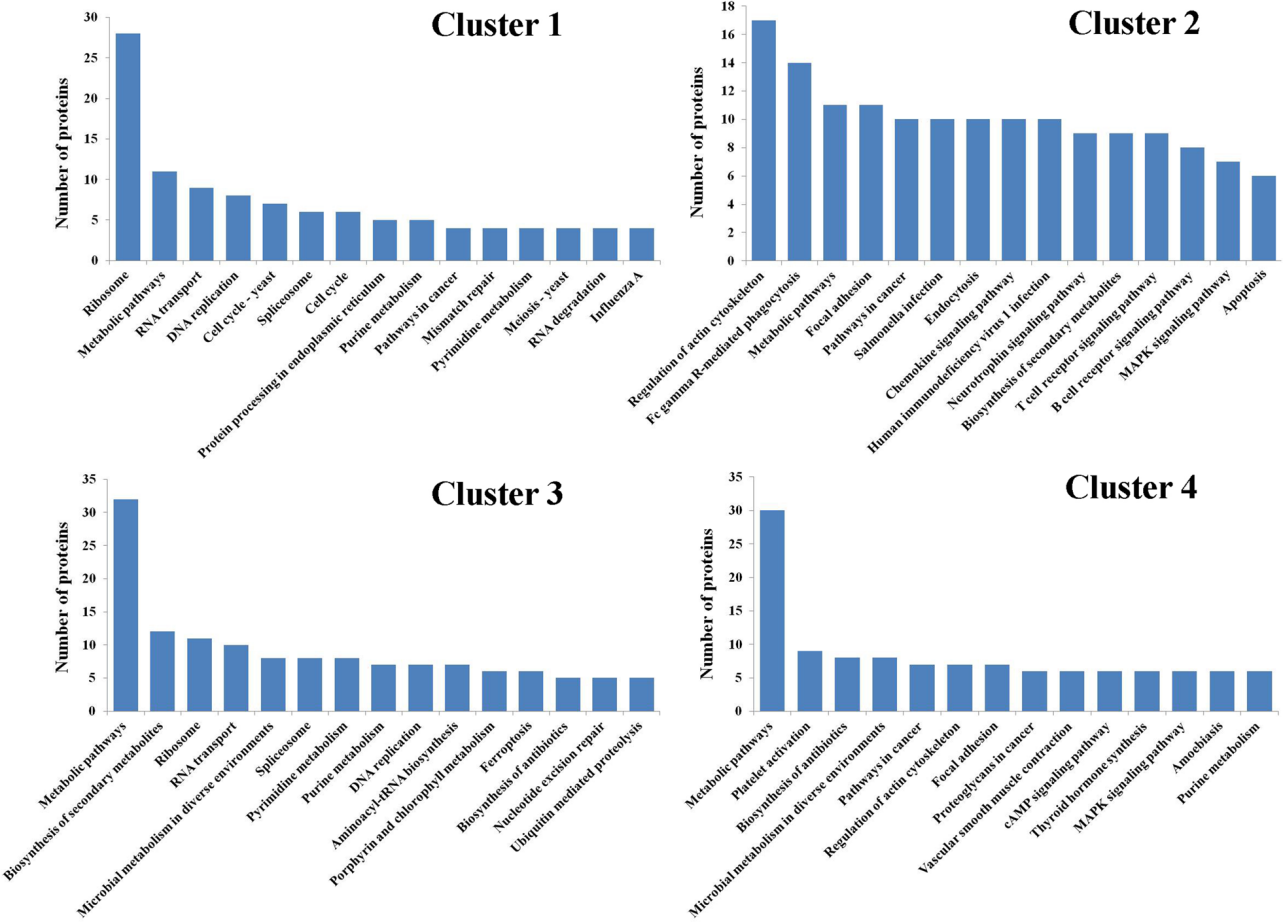
KEGG pathway analysis of the differentially expressed proteins in the four clusters

Proteins with different changing trends in the four clusters were also subject to KEGG signalling pathway analysis (Fig. 8). The results of the KEGG signalling pathway analysis for Cluster 2 and Cluster 4 are shown below. Expression levels of proteins in these 2 clusters had stable changing trends. In Cluster 2, 17 proteins were involved in the regulation of the actin cytoskeleton pathway (ko04810), suggesting that *Babesia* infection in host cells might induce changes in the host cytoskeleton in the early stage. Furthermore, we focused on pathways related to immunity and growth and development. The results showed that nine proteins were involved in the T cell receptor signalling pathway (ko04660), seven proteins were involved in the MAPK signalling pathway (ko04013), and six proteins were involved in the apoptosis pathway. In Cluster 4, six proteins were involved in the MAPK signalling pathway, and some proteins were involved in the iron metabolism pathway. These pathways play important roles in defending pathogen invasion in the body, regulating iron homeostasis and regulating growth and development in the body.

KEGG signalling pathway analysis was performed on 1011 phosphorylated proteins with phosphorylation modification changes. The results showed that these proteins were involved in a total of 174 signalling pathways (Additional file 1: Figure S5). Immune- and growth and development-related signalling pathways were enriched in different periods, indicating that immune regulation in the body plays important roles in the process of defending against *B. microti* infection. Related pathways involving immunity and growth and development include apoptosis (ko04210), ribosome (ko03010), MAPK signalling (ko04013) and T cell receptor signalling pathways (ko04660), and 14 proteins were involved in the apoptosis pathway, 13 proteins were involved in the T cell receptor signalling pathway, and 10 proteins were involved in the MAPK signalling pathway. In addition, proteins with different changing trends in the four clusters were also subject to KEGG signalling pathway analysis (Additional file 1: Figure S6).

### PRM to confirm the changes in the key proteins

The expression changes in ten key proteins at different infection stages were verified by PRM (Additional file 1: Figure S7). The quantitative results of the ten proteins were consistent with those shown by the DIA method [14, 15].

## Discussion

The spleen is the largest immune organ in mammals [16] and plays substantial roles in the activation and control of immune responses in the body [17, 18]. The spleen can kill a large number of invading pathogens during blood filtration and is the most important organ in the body for defending against *Babesia* infection [19, 20]. Although the spleen has a strong immune function, *Babesia* infection can also cause a series of morphological and physiological changes in the spleen [21]. In this study, we found that the subcellular structure of splenocytes from mice infected with *B. microti* also changed after in-depth exploration. For example, the endoplasmic reticulum expanded to varying degrees, which was accompanied by degranulation, and many lysosomes and autophagosomes appeared. During this process, the expression and modification of a large number of proteins in splenocytes changed. These proteins mainly participate in infection defence in the body, induction of apoptosis and autophagy, regulation of iron metabolism and cell proliferation and growth-related pathways (Table 1).

**Table 1:** Immune-related proteins, iron metabolism-related proteins and growth and development-related proteins in the spleen tissues of mice infected with *Babesia microti*

Protein name	Accession	Description	5 days	8 days	11 days	19 days
CTSD	P18242	Cathepsin D	↓	↓	↑	↑
IFI44	Q8BV66	Interferon-induced protein 44	↑	↑	–	–
ILF2	Q3UXI9	Interleukin-2 enhancer binding factor 2	↓	↓	↓	–
ILF3	A0A1L1STE4	Interleukin enhancer-binding factor 3	↓	↓	↓	–
STAT5A^a^	P42230	Signal transducer and activator of transcription 5A	–	↑	–	–
Lactoferrin	B8YJF9	Lactoferrin	↓	↓	↓	↓
Serotransferrin	Q921I1	Serotransferrin	↓	–	↑	–
TfR1	Q542D9	Transferrin receptor protein 1	↑	↑	↑	↑
GCLC	P97494	Glutamate-cysteine ligase	↑	↑	↑	↑
GCLM	O09172	Glutamate-cysteine ligase	–	–	↑	–
PKC-δ^a^	P28867	Protein kinase C-δ	–	↓	↓	–
MAPK1^a^	P63085	Mitogen-activated protein kinase 1	–	–	↓	–
MAPK3^a^	D3Z3G6	Mitogen-activated protein kinase 3	–	–	↓	–
Grb2	Q60631	Growth factor receptor-bound protein 2	↓	↓	↓	–
PAK2	Q8CIN4	P21-activated kinase 2	–	↓	↓	–

↑, Upregulated peptide; ↓, downregulated peptide; –, no change peptide

^a^Phosphorylated protein

### Immune-related proteins

The quantitative proteomics results in this study showed that the expression of many immune-related proteins changed in the mouse spleen during *B. microti* infection. These proteins mainly participate in physiological activities such as protein degradation in lysosomes, autophagy, apoptosis, inhibition of excessive cell proliferation and viral infection defence to ensure effective killing of *B. microti*.

After hosts are infected with common parasitic protozoans such as *Babesia*, *Plasmodium* and *Leishmania*, lysosomes in host cells can engulf a large number of parasites [22–24]. Cathepsin D (CTSD) belongs to the aspartic protease family. It is localized in lysosomes of various tissues and cells [25] and can participate in various physiological activities in cells including cell apoptosis [26], degrading exogenous pathogens through the autophagy-lysosome system [27] and promoting protein degradation in lysosomes during antigen presentation [28, 29]. This study showed that the expression level of CTSD was downregulated at 5 days and 8 days after *B. microti* infection and upregulated after 11 days. After the mice were infected with *B. microti*, the cell structure in spleen was destroyed; in addition, the structure and morphology of organelles such as lysosomes also had abnormalities. We speculate that this might be the cause of the corresponding reduction in the expression level of CTSD in the early stage of infection. With the decrease in the degree of infection, the cell structure in mouse spleen gradually returned to normal. At this time, lysosomes already engulfed a large number of *Babesia* and a large amount of CTSD was urgently needed for lysosomal degradation of *Babesia*. Therefore, the expression of level of CTSD was upregulated correspondingly.

Interferon-induced protein 44 (IFI44) is an interferon α/β-induced protein [30]. Studies have shown that IFI44 is a potential inflammatory factor and can defend against viral infection through the inhibition of viral transcription [31]. As an interferon-induced protein, IFI44 expression levels directly reflect IFN-α/β activity. IFN-α/β plays an important role in defending against infection from many parasites such as *Leishmania donovani* [32], *Plasmodium* [33], *Toxoplasma* [34] and *Trypanosome brucei* [35]. Therefore, IFI44 is speculated to have broad-spectrum anti-protozoan functions. This study showed that after mice were infected with *B. microti*, the expression level of IFI44 in the spleen was upregulated at 5 days and 8 days and was then gradually downregulated. The expression level returned to a normal level after 19 days. Therefore, we speculate that IFI44 participated in spleen immune responses after *B. microti* infection and directly or indirectly exerted its biological functions to defend against *B. microti* invasion. Although the mechanism of IFI44 involvement in defending against *B. microti* infection currently remains unclear, IFI44 might be able to be used as a marker for screening *Babesia* infection.

Interleukin-2 enhancer-binding factor 2 (ILF2) and interleukin enhancer-binding factor 3 (ILF3) are components of nuclear factor of activated T cells (NFAT) [36]. Downregulation of ILF2 expression levels has been shown to inhibit cell proliferation [37], whereas downregulation of IFL3 expression, in addition to inhibition of cell proliferation, also inhibits cell migration and invasion and promotes cell apoptosis [38]. Some parasites such as *Babesia*, *Plasmodium* and *Leishmania* can cause unlimited cell proliferation after infecting hosts to eventually cause diseases in hosts [39, 40]. This study showed that after *B. microti* infection in mice, the expression levels of ILF2 and IFL3 in the spleen were downregulated; notably, the expression levels were downregulated to the lowest levels at 11 days. The expression levels returned to normal at 19 days. Therefore, we speculate that *B. microti* infection in mice might result in excessive cell proliferation; therefore, reduced expression levels of ILF2 and IFL3 were required to inhibit unrestricted cell proliferation and avoid cell lesions.

The quantitative proteomics results for phosphorylated proteins in the spleen showed that phosphorylation modifications of some proteins changed after the spleen was infected by *Babesia*. These proteins were activated through changes in their phosphorylation modifications. STAT5 is an important transcription factor. After phosphorylation modification, signal transducer and activator of transcription 5A (STAT5A) can initiate target gene transcription to play a critical role in T cell proliferation and differentiation, thus participating in host immunomodulation [41]. The results of this study showed that the phosphorylation modification level of STAT5 in the spleens of mice after *B. microti* infection was upregulated at 8 days. We speculate that the main function of STAT5 activation was to promote T cell proliferation and differentiation [42] to ensure that more T cells participated in immune responses. With the gradual disappearance of *Babesia* under the clearance function of host immunity, the mouse spleen function and structure also gradually returned to normal at 19 days after infection, and the phosphorylation modification level of STAT5 also returned to a normal level. These results indicated that changes in the phosphorylation modification of STAT5 had important immune defence functions during the *Babesia* infection period, the synthesis of a large amount of proteins was not required, and the normal modification pattern rapidly returned to normal when it was not required in the body. This is a very energy-saving cascade signalling transduction process.

### Iron metabolism-related proteins

The iron ion is one of the important trace elements for the maintenance of life activities in the body [43]. Iron deficiency or excess can both cause adverse effects on health [44–49]. Many parasitic protozoans have the ability to influence iron metabolism in hosts such as *Plasmodium* and *Leishmania*. These parasites can take up a large number of iron ions in hosts to maintain their growth and propagation [50, 51]. When parasites propagate, they utilize hosts to provide enough iron supplements. It has been shown that when the iron content in hosts is too high, protozoan propagation indeed will be promoted [52]. In contrast, when the iron content in hosts is low, the parasitic rate significantly decreases. To maintain an iron element balance in the body, the body finely regulates iron absorption, transport and utilization.

Lactoferrin is an important non-haem iron-binding protein and participates in the regulation of iron homeostasis in the body [53]. In addition, as an immunomodulatory protein, lactoferrin has many functions, including anti-parasitic [54], antibacterial [55], anti-viral [56] and anti-inflammatory [57] actions. Lactoferrin participates in the host defence mechanism through two methods. The first method involves lactoferrin binding to iron in hosts so pathogens cannot acquire enough iron from host cells; therefore, their growth is blocked [58]. The other method involves lactoferrin directly interacting with pathogens to inhibit pathogen adsorption and invasion into target cells [56]. The results in this study showed that after *B. microti* infection in mice, the expression level of lactoferrin in splenocytes was downregulated during the infection period and upregulated during the recovery period. Therefore, we speculate that the reduction in the expression level of lactoferrin in the spleen in the infection period might be caused by the uptake of a large amount of iron in mice by *B. microti* to supply their growth needs. At this time, iron deficiency in the body caused excessive expression of iron transport-related proteins, thus decreasing the expression level of lactoferrin. However, with the extension of infection time, the iron content in hosts decreased dramatically. To maintain iron balance in the spleen during the recovery period, the uptake of iron by cells through various methods is needed. At this time, many lactoferrin proteins were required to assist in iron transport; therefore, the expression level of lactoferrin was upregulated.

Serotransferrin is a key protein involved in iron metabolism and in defending against microbial invasion in the body [59, 60]. It can not only transport iron in the body in a soluble and non-toxic form to participate in iron metabolism [61] but also inhibit the growth of pathogenic microorganisms through the clearance of free iron ions in hosts [60, 62]. The results of this study showed that the expression level of serotransferrin in the mouse spleen after *B. microti* infection was downregulated at 5 days and upregulated at 11 days. This regulation pattern for serotransferrin was similar to that for lactoferrin because they had similar functions in iron metabolism and anti-pathogen activities. They both have iron transport functions [63, 64]. In addition, lactoferrin also has iron ion-binding functions [54]. The coordinating regulation of these two proteins not only inhibited pathogen invasion in the body but also regulated iron balance in the body.

Transferrin receptor protein 1 (TfR1) distributes on the surfaces of mammalian cells to mediate the entry of iron taken up by transferrin from outside cells into cells [65]. The expression level of TfR1 has been shown to be negatively correlated with the iron reserve in the body [66]. When iron is deficient in the body, cells increase iron intake through the expression of high levels of TfR1 [47]. In contrast, when iron is excessive, the expression level of TfR1 will decrease correspondingly to decrease iron intake [67]. This study showed that after mice were infected with *B. microti*, the expression level of TfR1 was significantly upregulated in the mouse spleen, and the fold upregulation peaked at 11 days. With the gradual recovery of the body at 19 days, the expression level of TfR1 also recovered to a normal level. Therefore, we speculate that the characteristics of expression changes in TfR1 mainly reflect a dramatic reduction in iron reserves due to the consumption of a large amount of iron ions in mouse splenocytes by *B. microti* after mice were infected with *B. microti*. To maintain iron ion homeostasis, cells had to express high levels of TfR1 to accelerate intracellular iron ion intake.

Glutamate-cysteine ligase (GCL) is a rate-limiting enzyme of glutathione (GSH) synthesis [68]. It is a heterodimer composed of a modifier subunit (GCLM) and a catalytic subunit (GCLC) [69]. The results of this study showed that the expression of GCLM in the mouse spleen at 5 days after *B. microti* infection was continuously upregulated and returned to a normal level at 19 days, whereas GCLC expression was upregulated at 11 days and returned to a normal level at 19 days. These results indicated that the GCL expression level increased correspondingly after splenocytes were infected with *B. microti*. With the decrease in the infection level of *B. microti*, the GCL expression level also returned to a normal level. Infection of hosts by some common parasitic protozoans can promote an increase in oxygen free radical levels in the body and thus induce oxidative stress responses in the body [70]. GSH protects cells from oxidative damage [71], and the first step of GSH synthesis is catalysis by GCL [72]. Therefore, we speculate that the GCL expression level increased in the spleen after mice were infected with *B. microti* to effectively accelerate GSH biosynthesis. The presence of a large amount of GSH ensured high oxygen free radical clearance and antioxidant abilities [73] to maintain homeostasis in splenocytes.

### Growth and development-related proteins

This study showed that the expression levels or phosphorylation modification levels of many proteins involved in growth and development changed in mouse spleen during *B. microti* infection. Certain protozoan infections cause uncontrolled proliferation of host cells [74]. When the condition is severe, infection may even result in host organ failure until death [75]. To prevent excessive cell proliferation, the host body will adopt an effective response mechanism. Protein kinase C-δ (PKC-δ) is a Ser/Thr-specific kinase involved in many basic cellular processes, including growth and differentiation [76]. Under the function of many cytokines, including IFN-α, PKC-δ has been shown to be activated by phosphorylation. Activated PKC-δ can inhibit cell proliferation and promote cell apoptosis [77, 78]. This study showed that at 8 days and 11 days after *B. microti* infection in mice, both PKC-δ expression in the spleen and the phosphorylation modification level were downregulated. We speculate that this pattern of change in PKC-δ during the infection period prevented excessive proliferation of splenocytes during the *Babesia* infection period to avoid body damage.

MAPK is a protein kinase composed of Ser/Thr kinases [79]. The MAPK signal transduction pathway is linked with cell surface growth factors through growth factor receptor-bound protein 2 (Grb2) [80]. MAPK1 regulates cell proliferation, survival, adhesion and migration through the phosphorylation of hundreds of nuclear substrates and cytoplasmic substrates in cells [81]. MAPK3 also plays a critical role in cell proliferation [82]. We found that the phosphorylation modification level of MAPK1 was significantly downregulated at 11 days. The phosphorylation modification level of MAPK3 was significantly downregulated, and the fold downregulation was the highest at 11 days. Insufficient MAPK3/1 expression has been shown to block cell proliferation [82, 83]. Dephosphorylation of MAPK3/1 inhibits cell proliferation and differentiation [84]. In addition, this study showed that the expression level of Grb2 was downregulated after *B. microti* infection in mice and returned to a normal level at 19 days. The reduction in the Grb2 expression level reduced the abilities of various cytokines in response to the induction of proliferation signal transduction [85]. Therefore, we speculate that the regulation patterns of these MAPK signal transduction-related proteins in mouse spleen effectively inhibited the unrestricted proliferation of host cells during the infection period.

*Babesia* infection in host cells results in insufficient blood glucose in hosts [86]. P21-activated kinase 2 (PAK2) is an important participant in the insulin signalling pathway and glucose homeostasis [87]. Downregulation of PAK2 expression has been shown to promote glucose uptake [88]. This study showed that after *B. microti* infection in mice, the expression level of PAK2 in the spleen was downregulated at 8 days and 11 days and was close to a normal level at 19 days. We speculate that the blood glucose level decreased after *B. microti* infection in mice. Cells reduced the PAK2 expression level to promote glucose uptake to maintain glucose homeostasis.

## Conclusions

In this study, the roles of different proteins in the spleen in response to *B. microti* infection were analysed by high-throughput quantitative proteomics. Studies have shown that as the largest immune organ, the spleen responds to infections through specific immune-related proteins. At the same time, the spleen reduces the iron content in the body by regulating the expression levels of iron metabolism-related proteins, thereby reducing the degree of *B. microti* infection. The expression of some growth and development-related proteins was also reduced to inhibit excessive proliferation of cells, thereby preventing excessive damage to the spleen. By analysing the characteristics of these proteins, we can further understand the molecular regulatory mechanism of the spleen in response to infection, thus creating conditions for improving the diagnostic efficiency and treatment of babesiosis.

## Supplementary Information

**Additional file 1: Figure S1.** The whole experimental design for the proteomics analysis of global proteins and phosphorylated proteins in spleen tissues from mice infected with *B. microti.* The photos of the spleen show the spleen morphology in different stages. **Figure S2.** Statistics for the identified phosphorylated peptides. (a) Venn diagram of phosphorylated peptide fragments identified at all stages. (b) PCA of phosphorylated peptide mass spectrometry data at the 5 sampling times. **Figure S3.** Cluster analysis of differentially phosphorylated peptides in the spleen tissues of mice infected with *B. microti*. A log2 value > 0.58 indicated upregulation, showing that this peptide and its phosphorylation modification level were upregulated. A log2 value < − 0.58 indicated downregulation, showing that this peptide and its phosphorylation modification level were downregulated. **Figure S4.** GO functional annotations of differentially phosphorylated proteins. Biological Process, Cellular Component and Molecular Function (orange, green and blue bars, respectively). **Figure S5.** KEGG pathway analysis of the differentially phosphorylated proteins. **Figure S6.** KEGG pathway analysis of the differentially phosphorylated proteins in the four clusters. **Figure S7.** PRM analysis of the ten key proteins. The expression trends of ten key proteins and their corresponding peptides are basically the same.

**Additional file 2: Dataset S1.** Information for intersecting global proteins. **Dataset S2.** Information for intersecting phosphopeptides.

## Data Availability

Data supporting the conclusions of this article are included within the article. The mass spectrometry proteomics data have been deposited in the ProteomeXchange Consortium via the iProX partner repository (accession no. IPX0002204000/PXD019236; no. IPX0002209000/PXD019319).

## Abbreviations

DIA: Data-independent acquisition
CTSD: Cathepsin D
IFI44: Interferon-induced protein 44
ILF2: Interleukin-2 enhancer-binding factor 2
ILF3: Interleukin enhancer-binding factor 3
STAT5A: Signal transducer and activator of transcription 5A
TfR1: Transferrin receptor protein 1
GCL: Glutamate-cysteine ligase
PKC-δ: Protein kinase C-δ
MAPK: Mitogen-activated protein kinase
Grb2: Growth factor receptor-bound protein 2
PAK2: P21-activated kinase 2
TEM: Transmission electron microscope
ACN: Acetonitrile
TFA: Trifluoroacetic acid
RP-HPLC: Reversed phase high-performance liquid chromatography
DDA: Data-dependent acquisition
AGC: Automatic gain control
FDR: False discovery rate
PCA: Principal component analysis
GO: Gene Ontology
KEGG: Kyoto Encyclopedia of Genes and Genomes
PRM: Parallel reaction monitoring
AUC: Area under the curve
CV: Coefficient of variation
NFAT: Nuclear factor of activated T cells
GSH: Glutathione
GCLM: Glutamate-cysteine ligase modifier subunit
GCLC: Glutamate-cysteine ligase catalytic subunit
