# Supplementary material for: Protein regulation strategies of the mouse spleen in response to Babesia microti infection
Source: Parasit Vectors. 2021 Jan 19;14:61. doi: 10.1186/s13071-020-04574-5 (PMC7814643; doi:10.1186/s13071-020-04574-5)
Supplement: Supplementary file 1 — Additional file 1: Figure S1. The whole experimental design for the proteomics analysis of global proteins and phosphorylated proteins in spleen tissues from mice infected with B. microti. The photos of the spleen show the spleen morphology in different stages. Figure S2. Statistics for the identified phosphorylated peptides. (a) Venn diagram of phosphorylated peptide fragments identified at all stages. (b) PCA of phosphorylated peptide mass spectrometry data at the 5 sampling times. Figure S3. Cluster analysis of differentially phosphorylated peptides in the spleen tissues of mice infected with B. microti. A log2 value > 0.58 indicated upregulation, showing that this peptide and its phosphorylation modification level were upregulated. A log2 value < − 0.58 indicated downregulation, showing that this peptide and its phosphorylation modification level were downregulated. Figure S4. GO functional annotations of differentially phosphorylated proteins. Biological Process, Cellular Component and Molecular Function (orange, green and blue bars, respectively). Figure S5. KEGG pathway analysis of the differentially phosphorylated proteins. Figure S6. KEGG pathway analysis of the differentially phosphorylated proteins in the four clusters. Figure S7. PRM analysis of the ten key proteins. The expression trends of ten key proteins and their corresponding peptides are basically the same. [file 13071_2020_4574_MOESM1_ESM.docx]

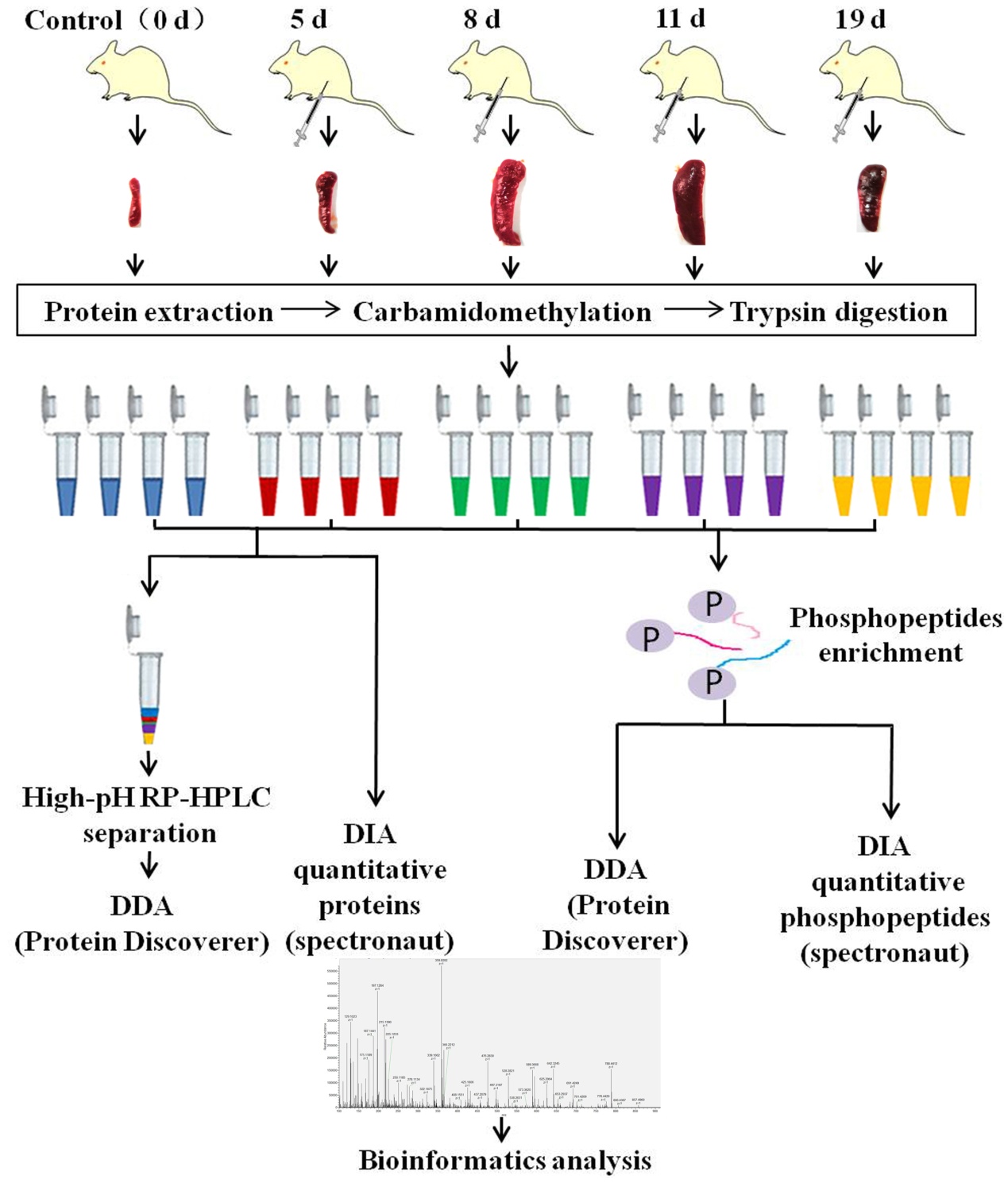


**Figure S1.** The whole experimental design for the proteomics analysis of global proteins and phosphorylated proteins in spleen tissues from mice infected with *B. microti.* The photos of the spleen show the spleen morphology in different stages.


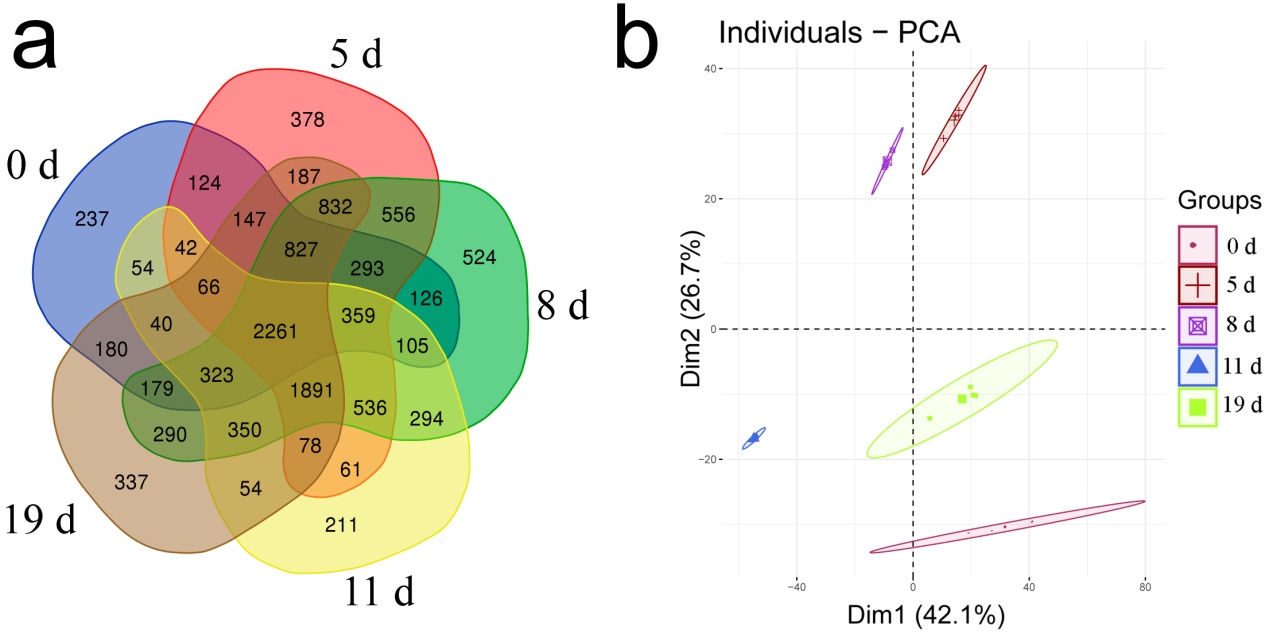


**Figure S2.** Statistics for the identified phosphorylated peptides. (a) Venn diagram of phosphorylated peptide fragments identified at all stages. (b) PCA of phosphorylated peptide mass spectrometry data at the 5 sampling times.


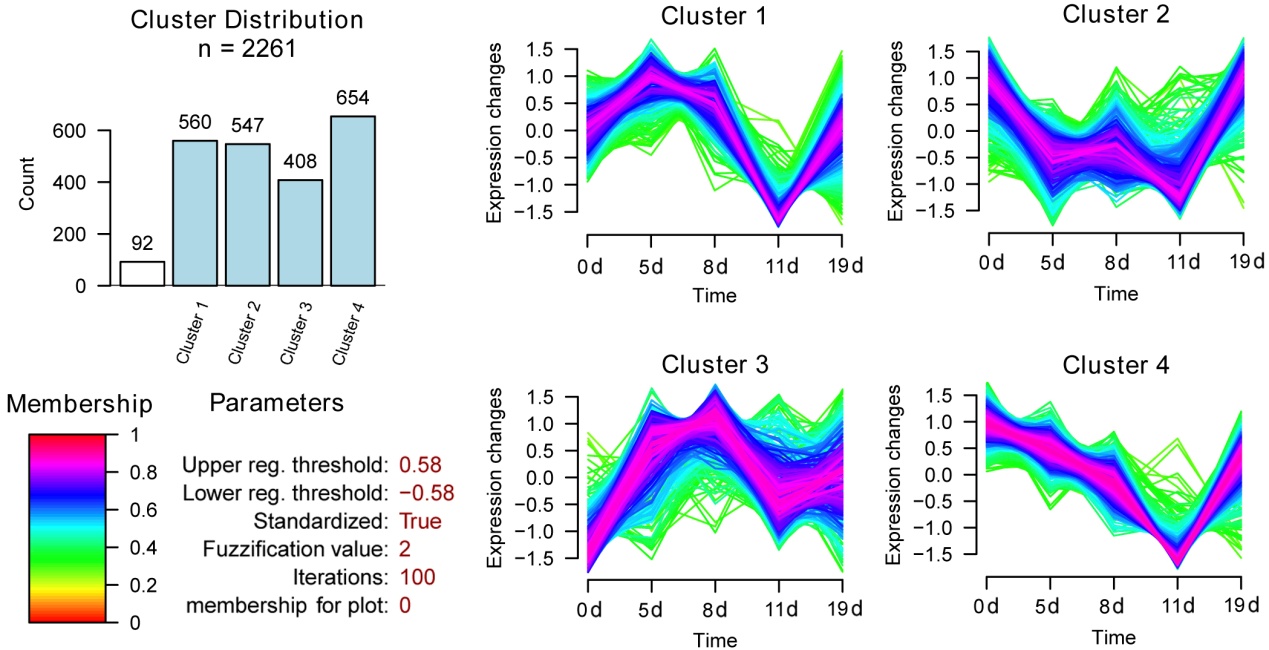


**Figure S3.** Cluster analysis of differentially phosphorylated peptides in the spleen tissues of mice infected with *B. microti*. A log2 value >0.58 indicated upregulation, showing that this peptide and its phosphorylation modification level were upregulated. A log2 value <–0.58 indicated downregulation, showing that this peptide and its phosphorylation modification level were downregulated.


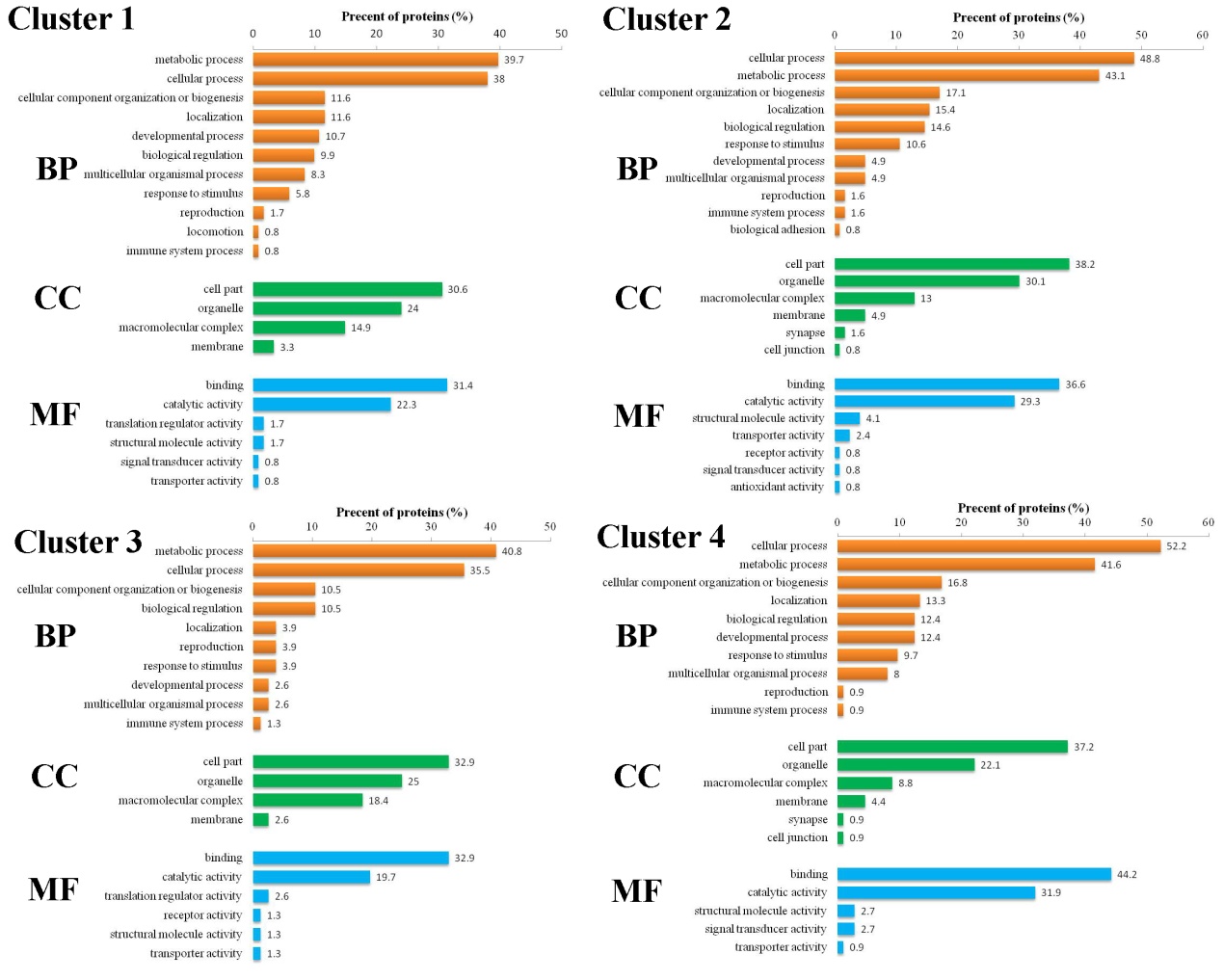


**Figure S4.** GO functional annotations of differentially phosphorylated proteins. Biological Process, Cellular Component, and Molecular Function (orange, green, and blue bars, respectively).


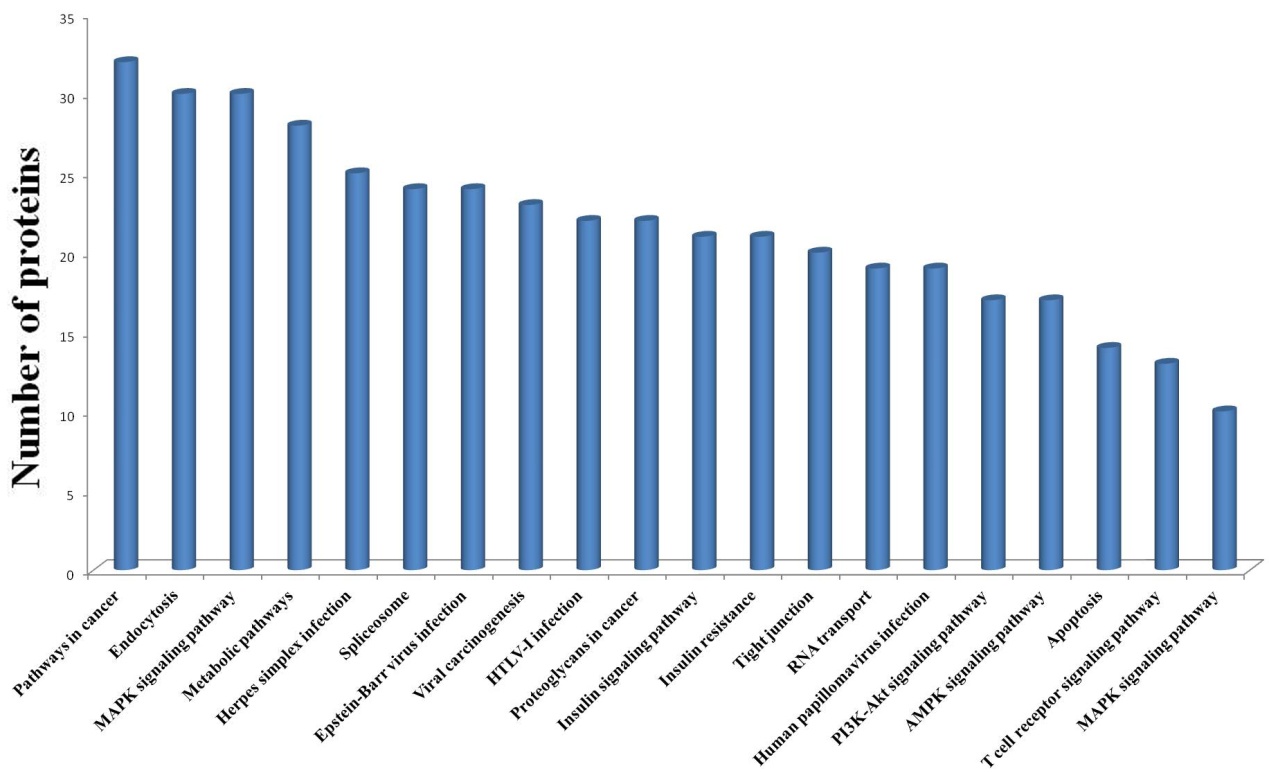


**Figure S5.** KEGG pathway analysis of the differentially phosphorylated proteins.


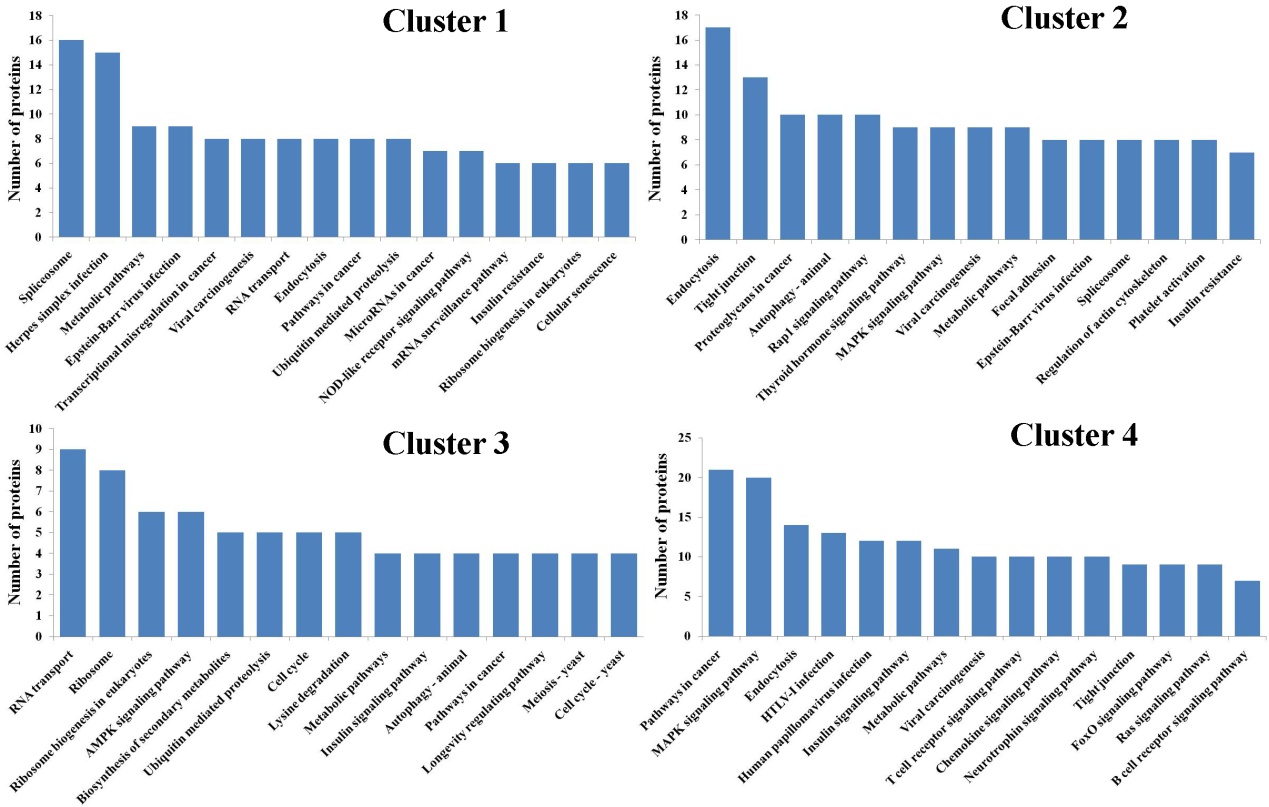


**Figure S6.** KEGG pathway analysis of the differentially phosphorylated proteins in the 4 clusters.


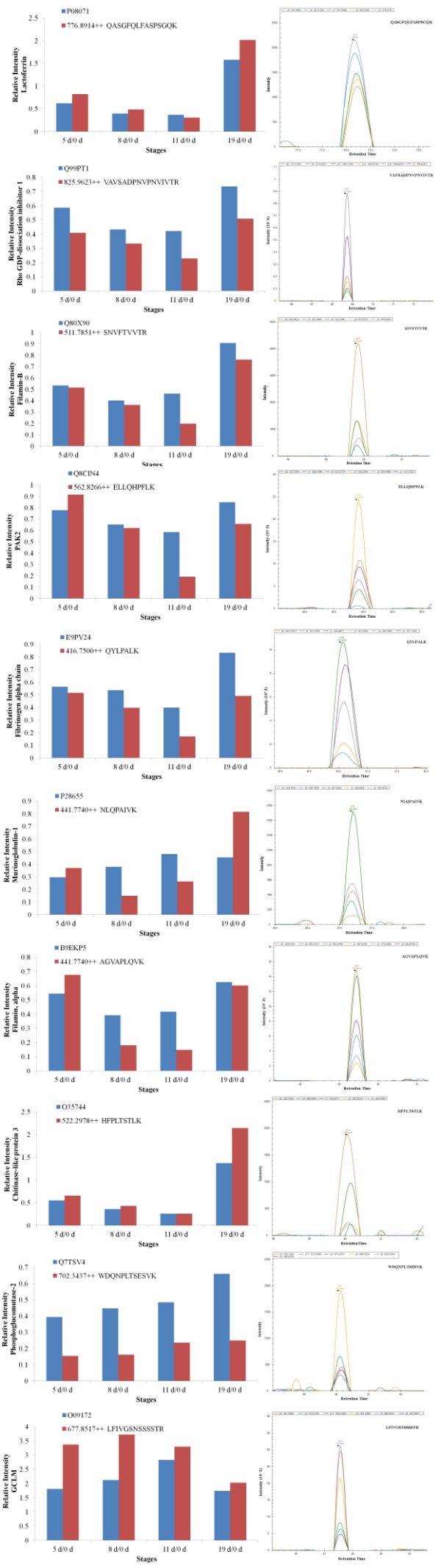


**Figure S7.** PRM analysis of the 10 key proteins. The expression trends of 10 key proteins and their corresponding peptides are basically the same.
